# Laparoscopic Optimal Excision of Deep Rectovaginal Endometriosis: Tips and Techniques

**DOI:** 10.7759/cureus.84239

**Published:** 2025-05-16

**Authors:** Shweta More, Kunal Rathod

**Affiliations:** 1 Obstetrics and Gynaecology, Queen’s Hospital, King George Hospitals, Barking, Havering and Redbridge University Hospitals NHS Trust, London, GBR

**Keywords:** deep rectovaginal endometriosis, discoid bowel resection, laparoscopy, ovarian suspension, ureterolysis

## Abstract

Endometriosis is a chronic progressive disease spectrum characterized by the presence of tissue resembling functioning endometrial glands and stroma outside the uterine cavity. These ectopic implants have a propensity to bleed, initiating an inflammatory response. One of the most severe forms is deep rectovaginal endometriosis, which is the most challenging entity to treat. The diagnostic evaluation of these patients requires a transvaginal ultrasound and an MRI pelvis. Medical management may reduce the symptom severity but does not cure the disease. These patients often need surgical excision of the endometriosis to manage symptoms. We aim to describe the approach, principles, and detailed techniques of surgical removal of deep endometriosis. Complete excision of the deep rectovaginal endometriosis was achieved with symptomatic relief. This case illustrates our presurgical evaluation and operative techniques in detail, which are crucial in the optimal removal of endometriosis.

## Introduction

Endometriosis is a chronic progressive, clinical, and pathologic entity characterized by the presence of tissue resembling functioning endometrial glands and stroma outside the uterine cavity, affecting 7-11% of women of the reproductive age group [1]. These ectopic endometrial implants have a propensity to bleed under the influence of hormonal changes, and this triggers the inflammatory response in the adjacent areas. This inflammatory response is considered an important factor in the formation of pelvic adhesions. These adhesions can range from flimsy, thin, translucent to a severe condition called a frozen pelvis [1,2]. It presents in three distinct categories, like peritoneal, ovarian, and deep pelvic endometriosis. Deep endometriosis (DE) is identified when there is peritoneum infiltration by endometriotic tissue, more than 5 mm [3]. The various locations for the pathology could be involving uterosacral ligament, bowel, bladder, ureter, vagina, parametrium, and the diaphragm.

Deep pelvic endometriosis is the most challenging entity to treat. Medical management may reduce the symptom severity but does not cure the disease. These patients often need surgical excision to manage symptoms of pain, subfertility, sexual problems, and bowel or urinary symptoms [4]. Surgical management of endometriosis involves both minimally invasive, laparoscopic, and robotic excision and open surgical excision. However, laparoscopy has become the standard of care over the years in comparison to open surgery [5].

The basic principles of endometriosis excision surgery remain the same regardless of the surgical approach and location of the disease. Sound and safe operating techniques are crucial in the management and optimal removal of endometriosis. Dissection of the avascular spaces in the pelvis is a key for safe endometriosis surgery [5,6]. We aim to provide detailed surgical techniques for the excision of deep pelvic endometriosis.

## Technical report

Case details

**Figure 1:**
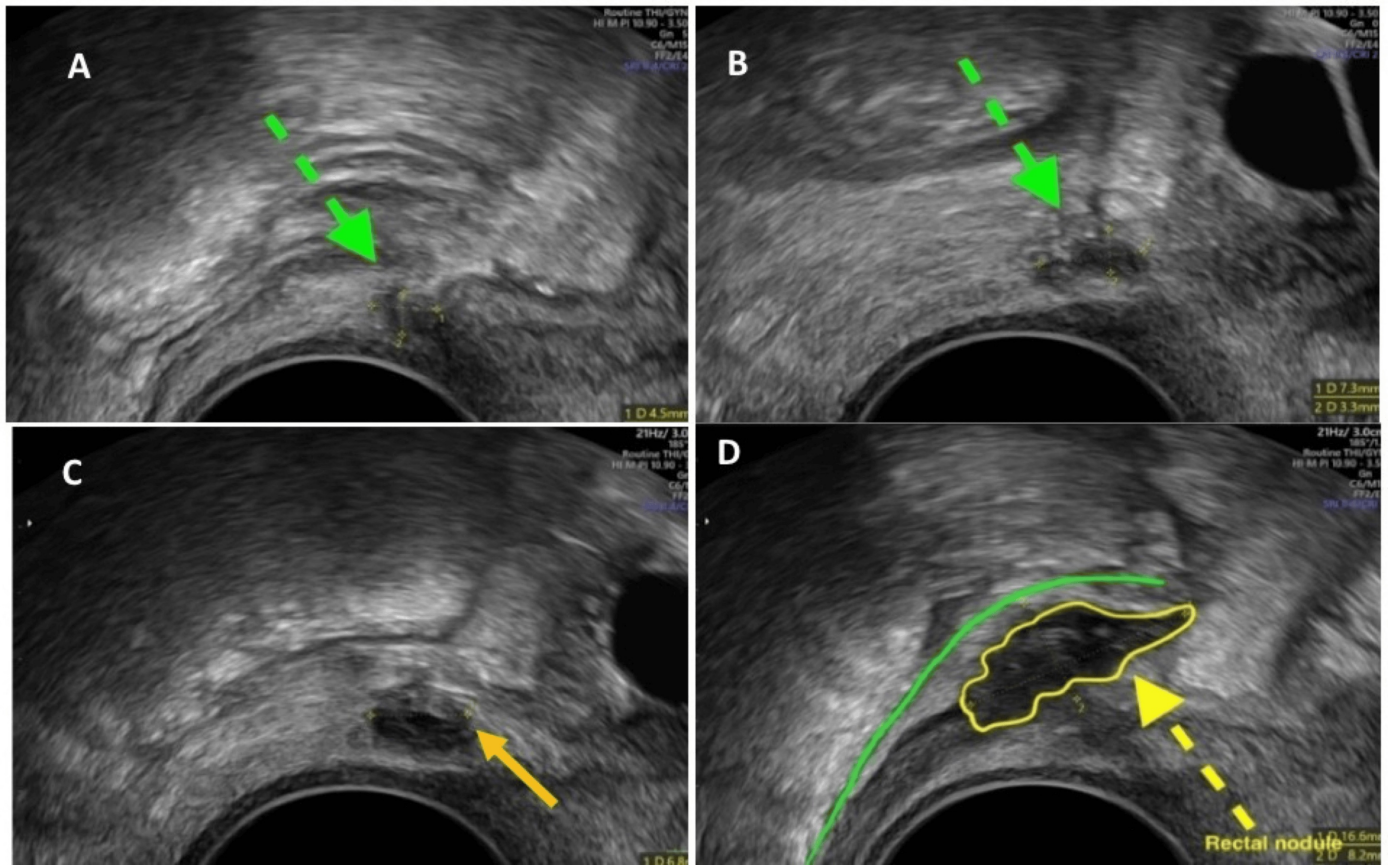
A) TVS ultrasound image showing an endometriotic nodule in the right uterosacral ligament (green dotted arrow). B) TVS ultrasound image showing an endometriotic nodule in the left uterosacral ligament (green dotted arrow). C) Endometriosis nodule in the right parametrium (yellow arrow). D) Endometriosis nodule along the anterior wall of the upper rectum (yellow arrow, green line outlines the mucosa of the rectum). TVS: transvaginal

A 31-year-old female had symptoms of dysmenorrhea, dyschezia, dyspareunia, and chronic pelvic pain. She received progesterone hormonal treatment in the past without any significant relief. Transvaginal (TVS) ultrasound pelvis was performed, which showed endometriotic nodules affecting the bilateral uterosacral ligament, pouch of Douglas, and right parametrium (Figure 1A-1D). There was also an endometriotic nodule affecting the anterior wall of the upper rectum 12 cm from the anal verge, confined to the muscularis layer. 

Pre-surgical evaluation/approach

The clinical history and previous medical and surgical treatments related to endometriosis need to be recorded in detail. Therapeutic planning depends on many factors, including the age of the patient, her desire for fertility or pain relief, the duration and intensity of her symptoms, the extent of disease, and previous treatments that have been undertaken.

The endometriosis-focused multidisciplinary team discussion with radiology and colorectal team helped us to plan the safe surgical approach. Preoperative TVS ultrasound and MRI pelvis imaging were done to understand the extent of the disease to tailor the surgical planning. Preoperative bowel preparation is essential in all cases. 

Stepwise description of surgical techniques

Adequate Exposure of the Operative Field

**Figure 2:**
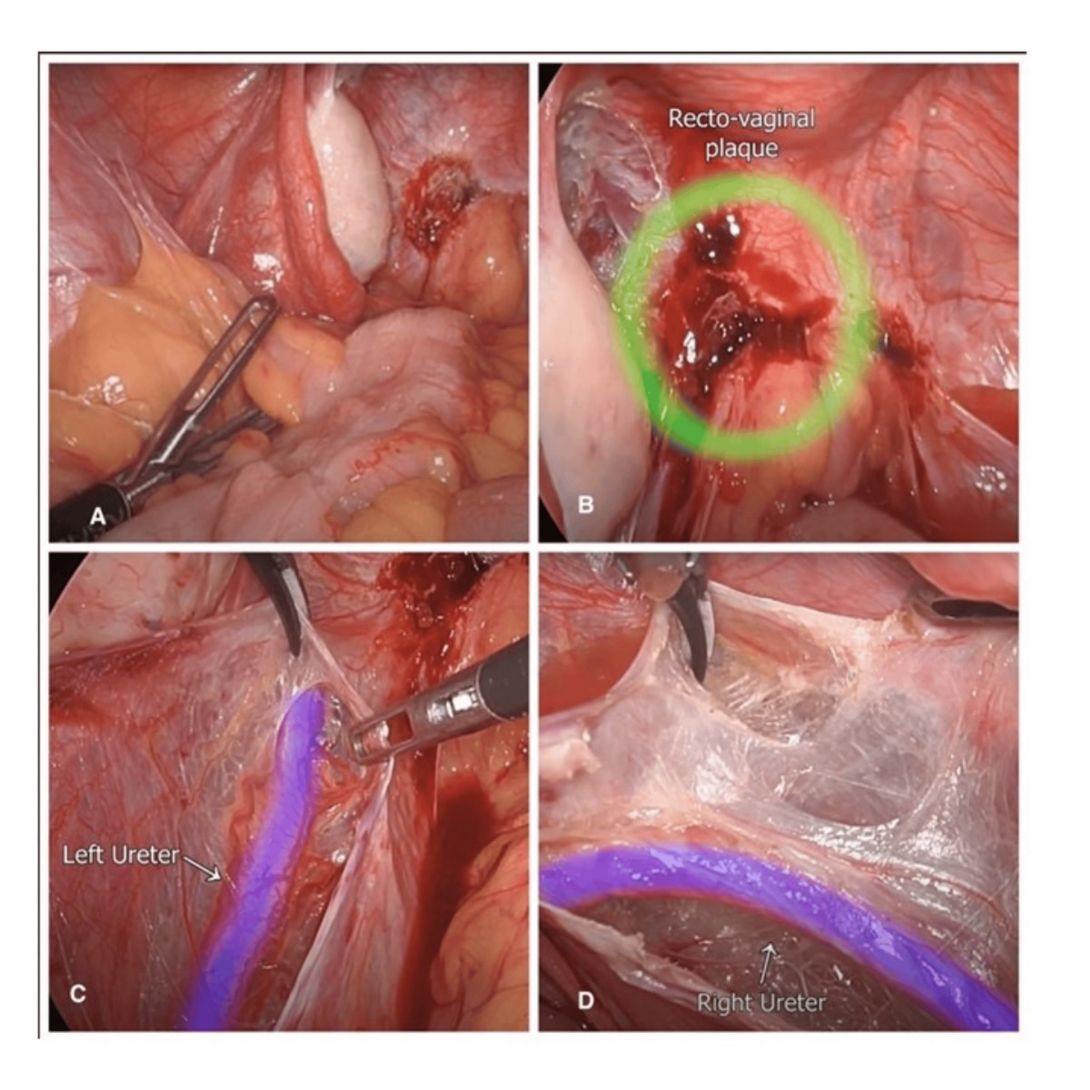
A) Mobilization of the sigmoid colon from its embryonic attachment to the lateral pelvic wall. B) Rectovaginal endometriotic nodule obliterating the pouch of Douglas (green circle). C) Dissection of the left ureter along with its intact vasculature. D) Medial deviation of the right ureter changing its course.

The mobilization of the sigmoid colon from its embryonic attachment to the lateral pelvic wall was done to achieve clear operative visualization of the pelvic cavity, to prevent inadvertent injury to bowel loops, and to facilitate left ureterolysis (Figure 2A). The gentle medial and caudal traction by the grasper led to the visualization of the white line of Toldt. The process of the bowel loop mobilization was initiated at the most distal site of the sigmoid colon, which is usually located at the pelvic brim. Following the retraction of the bowel, a peritoneal incision is made lateral and parallel to the descending colon (Figure 2B).

Ureterolysis

**Figure 3:**
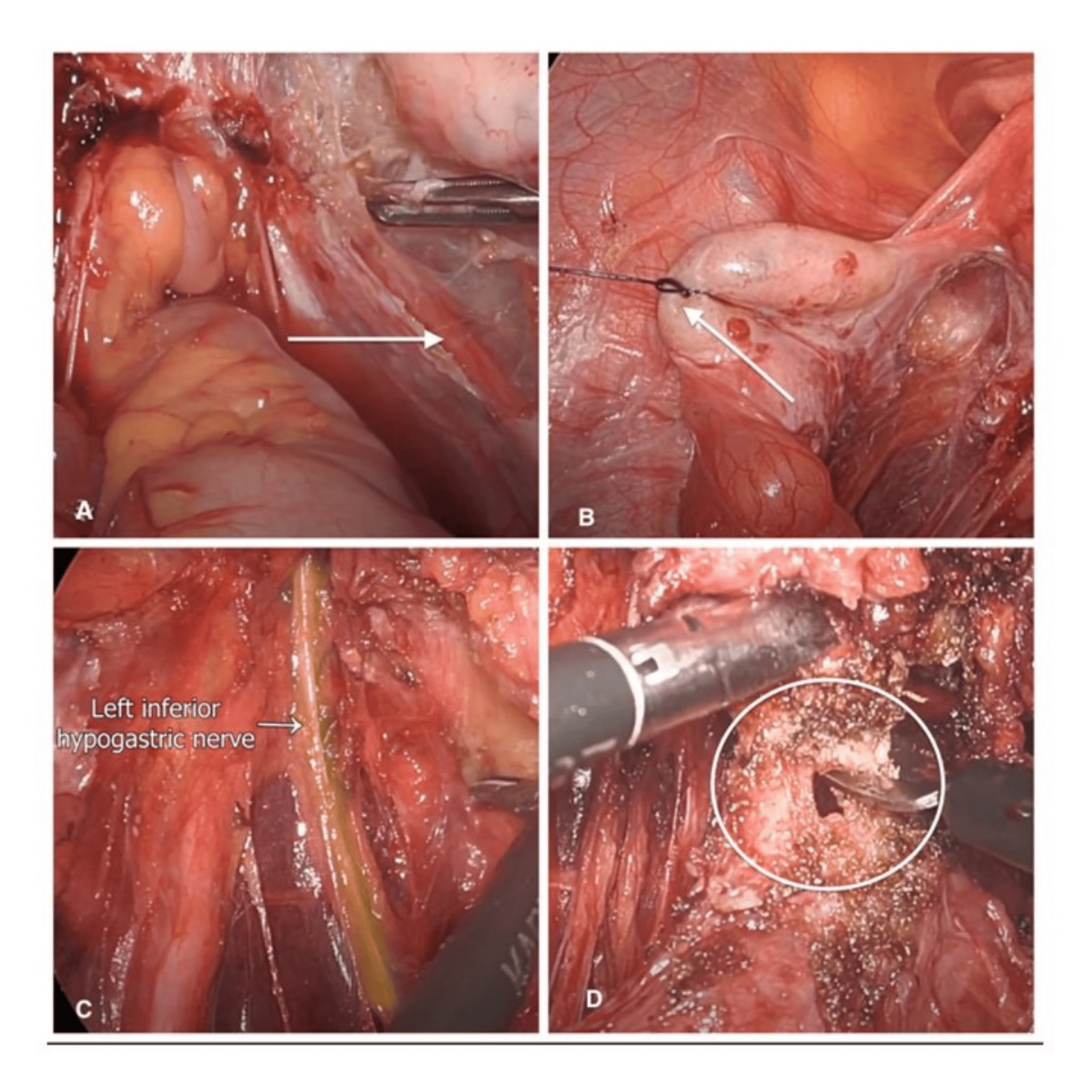
A) Medial deviation of the right ureter secondary to fibrosis of the rectovaginal endometriosis nodule (white arrow). B) Bilateral ovaries (white arrow) suspended to the anterior abdominal wall. C) Pararectal avascular space dissection to mobilize the segment of the rectum and hypogastric nerve course (white arrow). D) Discoid resection of the rectum (white circle) due to partial thickness involvement.

The aim of ureterolysis is to lateralize the ureter, which prevents injury to it during the surgery by keeping it under the direct vision of the surgeon. It also acts as an important anatomical landmark to prevent large vessel injury on the lateral pelvic wall [7]. The dissection of the ureter was started at the plane where the ureter enters the pelvic brim. A tent and incision were made in the normal peritoneum adjacent to the involved area. The inferior margin of the incision was grasped and deviated medially, and the ureter was separated from the peritoneum bluntly (Figure 2C). In this scenario, the right ureter deviated medially toward fibrosed adipose tissue in the pararectal space and deep rectovaginal endometriosis complex (Figure 2D). A careful dissection and lateralization of the right ureter was performed to reduce the risk of ureteric injury (Figure 3A). The important aspect of ureterolysis is to avoid the use of an energy source close to the ureter to maintain its blood supply. Peri-ureteral vessels in the adventitial layer must remain intact to prevent ischemia and resultant fistula formation [7].

Ovarian Suspension

The suture-mediated elevation of both ovaries was performed to the anterior pelvic wall using synthetic Stratafix 2-0 on a round body needle (Figure 3B). This allowed access to the ovarian fossa and lateral pelvic wall. It aids in maintaining the traction during dissection, which frees the hands of the operating surgeon/assistant to perform other tasks [8]. These sutures were removed immediately after the procedure was completed. 

Pararectal and Presacral Space Dissection

The pararectal and presacral space is an avascular space that provides access to rthe ectovaginal pouch and the dorsal aspect of the rectum to achieve complete excision of the disease [9]. The use of a rectal probe can facilitate the pararectal space. Careful dissection was performed to prevent injury to the hypogastric nerve plexus (Figure 3C).

Discoid Resection of the Bowel

If the endometriosis involves the partial thickness of the rectum, less than 3 cm, and a unifocal lesion, dissection of the nodule can be achieved by discoid resection of the bowel [10,11]. Discoid resection can be achieved by cutting peri-nodular with a scissor or circular stapler (Figure 3D). After resection, the bowel edge was approximated at the right angle to the long axis of the bowel to avoid the tension on the sutures and stricture of the bowel. The bowel integrity was confirmed by performing the leak test at the end of the procedure [12,13]. This test is performed by submerging the bowel loops into a pool of clear saline in the POD. We checked for any air bubbles after putting air through the rectum. In the absence of any visible air bubbles, the test confirms the bowel integrity.

Post-operative follow-up

The post-operative period was uneventful, and the patient was discharged after 24 hours with analgesic and laxative support. Clinical follow-up was done at two weeks and eight weeks at the gynecology and colorectal surgery clinic. It showed improvement in the symptoms with normal bowel and bladder function. The histopathology confirmed endometriosis of the area and two-thirds involvement of the rectal wall without mucosal involvement.

## Discussion

Deep rectovaginal endometriosis is one of the most severe forms of endometriosis and affects between 3.8% and 37% of all patients with endometriosis [1,2]. It is considered a stage four according to the American Society of Reproductive Medicine (ASRM) classification [3]. Patients usually present with symptoms of chronic pelvic pain, especially around the menstrual period, dyschezia, or severe dyspareunia. Further approach in these suspected patients involves clinical examination paired with diagnostic imaging, like transvaginal ultrasound and MRI pelvis. Diagnostic imaging plays a vital role in surgical planning. Medical management can give temporary relief from symptoms while waiting for surgery. These patients often need education and support for their emotional and psychological well-being. Surgery for recto-vaginal endometriosis is associated with plenty of technical challenges and calls for thorough evaluation [4,5].

These patients should be treated in specialized centers under a multidisciplinary team approach. Preoperative planning, opening pelvic avascular spaces during the dissection, and a stepwise systematic approach are key for successful surgery [6,7]. Endometriosis lesions with superficial involvement of the rectal wall are preferably treated with local laparoscopic excision in the form of shaving, while segmental rectal resection is needed in the case of severe, circumferential infiltration with stenosis. For intermediate disease, the current practice is discoid resection [10-12]. The recurrence rate of endometriosis is considerably lower with segmental resection and discoid excision than with rectal shaving [12,13]. 

Recent studies have demonstrated the importance of the nerve-sparing laparoscopic approach while performing excision for DE [14]. It helps to preserve the bowel, bladder, and sexual function by avoiding iatrogenic injury to pelvic autonomic nerves. The careful dissection of the presacral and pararectal space is helpful to prevent iatrogenic injury to pelvic autonomic nerves [14,15].

## Conclusions

The laparoscopic approach for deep endometriosis demands high-level surgical skills, which has a long learning curve but provides superior visualization of the posterior cul-de-sac. The surgical approach allows a high degree of magnification of peritoneal surfaces, which aids in the identification of subtle lesions and also provides a detailed anatomical view of blood vessels, nerves, and the ureter. This leads to precise excision of the endometriotic nodules. Good preoperative workup with transvaginal ultrasound and MRI pelvis, along with a focused multidisciplinary approach, aids in surgical planning for best results. Optimal excision of the endometriosis has a significant impact on the patient’s quality of life as it improves the pain.

In our experience, a stepwise approach during the laparoscopic surgery of deep endometriosis gives the best outcome and reduces the surgical morbidity. This stepwise surgical approach is a reproducible skillset that can be transferred to junior laparoscopic surgeons while dealing with deep endometriosis surgery.
